# Predicting postoperative malnutrition in patients with oral cancer: development of an XGBoost model with SHAP analysis and web-based application

**DOI:** 10.3389/fonc.2025.1564459

**Published:** 2025-05-12

**Authors:** Lixia Kuang, Jingya Yu, Yunyu Zhou, Yu Zhang, Guangman Wang, Fangmin Zhang, Grace Paka Lubamba, Xiaoqin Bi

**Affiliations:** ^1^ State Key Laboratory of Oral Diseases & National Center for Stomatology & National Clinical Research Center for Oral Diseases & Department of Orthognathic and Temporomandibular Joint (TMJ) Surgery, West China Hospital of Stomatology, Sichuan University, Chengdu, China; ^2^ Department of Hepatobiliary, Chongqing Fuling Hospital, School of Medicine, Chongqing University, Chongqing, China; ^3^ West China School of Nursing, Sichuan University, Chengdu, China; ^4^ School of Stomatology, North Sichuan Medical College, Nanchong, Sichuan, China; ^5^ State Key Laboratory of Oral Diseases & National Center for Stomatology & National Clinical Research Center for Oral Diseases & Department of Head and Neck Oncology, West China Hospital of Stomatology, Sichuan University, Chengdu, China; ^6^ Department of Oral and Maxillofacial Surgery, University Clinics of Kinshasa, Faculty of Dental Medicine, University of Kinshasa, Kinshasa, Democratic Republic of Congo

**Keywords:** malnutrition, oral neoplasms, machine learning, Shapley Additive exPlanations (SHAPs), postoperative complications

## Abstract

**Background:**

Postoperative malnutrition, which significantly affects recovery and overall quality of life, is a critical concern for patients with oral cancer. Timely identification of patients at nutritional risk is essential for implementing appropriate interventions, thereby improving postoperative outcomes.

**Methods:**

This prospective study, which was conducted at a tertiary hospital in China between August 2023 and May 2024, included 487 postoperative oral cancer patients. The dataset was divided into a training set (70%) and a validation set (30%). Predictive models were developed via four supervised machine learning algorithms: logistic regression (LR), support vector machine (SVM), light gradient boosting machine (LGBM), and extreme gradient boosting (XGBoost). Nutritional risk was assessed via the Nutritional Risk Screening 2002 (NRS-2002) tool and diagnosed via the Global Leadership Initiative on Malnutrition (GLIM) criteria. Model performance was evaluated on the basis of discrimination, calibration, and clinical applicability, with SHAP analysis used for interpretability. Statistical analysis was conducted via R software, with appropriate tests for continuous and categorical variables.

**Results:**

Of the 487 oral cancer patients, 251 (51.54%) experienced postoperative malnutrition. The study cohort was split into a training set comprising 340 patients and a validation set comprising 147 patients. Seven key predictors were identified, including sex, T stage, repair and reconstruction, diabetes status, age, lymphocyte count, and total cholesterol (TC) level. The XGBoost model demonstrated an area under the curve (AUC) of 0.872 (95% CI: 0.836–0.909) in the training set and 0.840 (95% CI: 0.777–0.904) in the validation set. Calibration curves confirmed the model’s robust fit, and decision curve analysis (DCA) indicated substantial clinical benefit.

**Conclusion:**

This study represents the first development of an XGBoost-based model for predicting postoperative malnutrition in patients with oral cancer. The integration of SHAP for model interpretability, along with the creation of an intuitive web tool, enhances the model’s clinical applicability. This approach can significantly reduce malnutrition-related complications and improve recovery outcomes for oral cancer patients.

## 1 Introduction

Oral cancer ranks among the most prevalent malignant tumors in the head and neck region (1), encompassing cancers that develop at various anatomical sites, including the tongue, cheeks, gums, hard palate, and floor of the mouth (2). According to global statistics, approximately 377,000 new cases of oral cancer are diagnosed globally each year, with approximately 50,000 of these cases occurring in China (3). Surgical intervention remains the primary treatment for oral cancer. However, owing to the unique anatomical location of the tumor, the metabolic changes associated with malignancy, and the functional impairments following surgery, patients are particularly vulnerable to malnutrition postoperatively. This condition is often compounded by nutrition-related symptoms, which can significantly affect the quality of life of oral cancer patients following surgery (4, 5).

Malnutrition is a critical concern among patients with head and neck cancer (HNC), with studies indicating that 46–49% of these individuals face a significantly greater risk of severe malnutrition than do those with other malignancies (6). The global burden of oropharyngeal cancer continues to rise, and malnutrition, along with associated nutritional deficiencies, represents a major contributor to the disease burden in patients with head and neck tumors (7). Postoperative malnutrition, in particular, is strongly associated with prolonged hospital stays, higher complication rates, diminished treatment efficacy, and delayed recovery among oral cancer patients (8–10). To address these challenges, effective nutritional risk screening and assessment are therefore essential components of perioperative care, forming the foundation of comprehensive nutritional support therapy. Timely identification of patients at high nutritional risk, combined with early nutritional interventions, can significantly enhance postoperative recovery and improve clinical outcomes.

Recent advancements in artificial intelligence (AI) technology have accelerated its application in the biomedical field, offering innovative solutions to complex clinical challenges (11). Machine learning (ML) models have demonstrated remarkable accuracy in predicting various diseases and clinical conditions (12). The incorporation of interpretability methods, such as Shapley Additive exPlanations (SHAP), offers valuable insights into the interpretability of prediction results generated by ML models, ensuring transparency and clinical applicability (13). In this study, we aimed to develop and validate an interpretable ML model for the early and precise prediction of postoperative malnutrition risk in patients with oral cancer. Additionally, the model will utilize SHAP to identify and explain the significance of predictive features, enhancing its usability for clinical decision-making. This approach seeks to facilitate efficient nutritional risk management, ultimately improving postoperative outcomes and advancing the quality of care for individuals with oral cancer.

## 2 Methods

### 2.1 Study design

**Figure 1:**
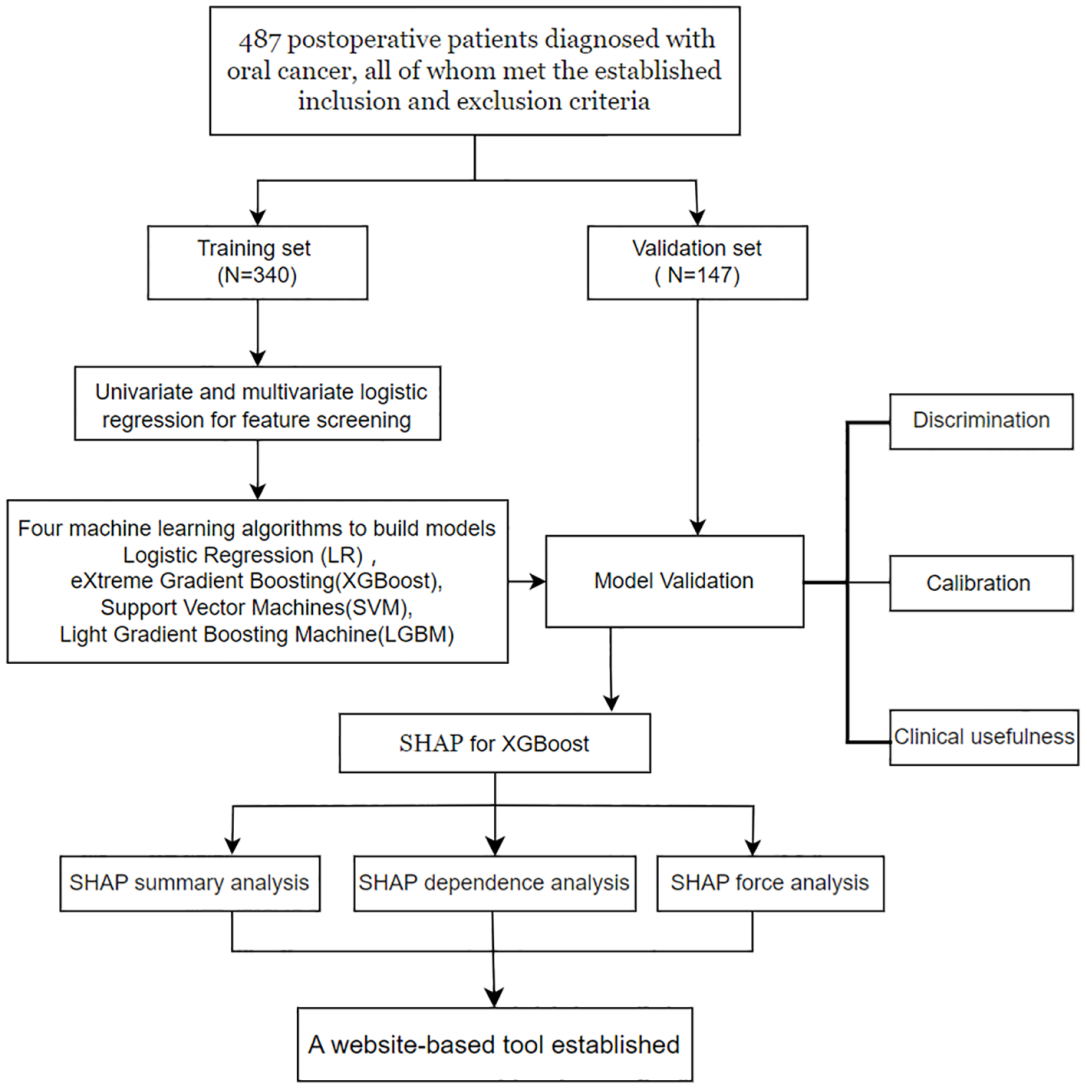
A schematic diagram of model development.

A prospective study involving 487 postoperative patients diagnosed with oral cancer from the Department of Head and Neck Oncology was conducted between August 5, 2023, and May 25, 2024, at the West China Hospital of Stomatology. This study employed a cross-sectional design to collect data, ensuring that all patient information was deidentified before being shared with the investigators to maintain confidentiality. To facilitate model development and validation, the dataset was randomly divided into two subsets at a 7:3 ratio: a training set and a validation set. The training set was used to develop predictive models via various machine learning algorithms, whereas the validation set was employed to evaluate the models’ performance. A detailed summary of the study procedures is presented in **Figure 1**.

### 2.2 Study population

This study included 487 postoperative patients who were diagnosed with oral cancer and were selected on the basis of well-defined inclusion and exclusion criteria. The inclusion criteria were as follows: (1) age ≥ 18 years, (2) confirmed pathological diagnosis of oral cancer with surgical treatment, (3) no malnutrition risk identified through preoperative nutritional risk screening, and (4) clear consciousness of the ability to read and understand study-related information. The exclusion criteria were (1) a prior diagnosis of a psychiatric disorder and (2) evidence of preoperative malnutrition.

### 2.3 Data collection

The clinical data of the participants were collected prospectively by the researchers through a detailed review of medical records. To ensure patient privacy, all the data were processed anonymously before analysis. The collected data included the following: (1) demographic characteristics, including sex, age, and body mass index (BMI); (2) tumor-related details, such as T stage, tumor site, tumor-related factors, and recurrence; (3) surgical information, including procedures such as cervical lymphatic dissection, repair and reconstruction, and tracheotomy; (4) participants’ medical history, including smoking, alcohol use, hypertension, and diabetes; and (5) preoperative laboratory test results, including erythrocyte, leukocyte, neutrophil, monocyte, and lymphocyte levels. All the data were systematically recorded to ensure accuracy and completeness.

### 2.4 Nutritional risk screening and nutritional assessment

Malnutrition was diagnosed via the Global Leadership Initiative on Malnutrition (GLIM) criteria through a two-step approach (14). On the seventh postoperative day, patients first underwent screening with the Nutritional Risk Screening 2002 (NRS-2002) tool to assess nutritional risk (15). An NRS-2002 total score of less than 3 indicated the absence of nutritional risk, whereas a score of 3 or higher signified the presence of nutritional risk. Patients with an NRS-2002 score ≥ 3 proceeded to the next step of malnutrition diagnosis, which involved evaluating three phenotypic criteria (nonvolitional weight loss, low BMI, and reduced muscle mass) and two etiologic criteria (reduced food intake or assimilation and disease burden or inflammation).

For patients with oral cancer, the disease burden was automatically considered to meet one of the etiologic criteria for malnutrition. BMI thresholds were adjusted for age: in patients over 70 years, a BMI below 22 kg/m² was classified as low, whereas for those under 70 years, a BMI below 20 kg/m² indicated low BMI (16). Nonvolitional weight loss was defined as an unintentional reduction in body weight exceeding 5% within the preceding six months or more than 10% over a period longer than six months (17). Reduced muscle mass was determined on the basis of grip strength, with cutoff values defined by the Asian Working Group for Sarcopenia: <18 kg for females and <26 kg for males (18). Patients were diagnosed with GLIM-defined malnutrition if they met at least one phenotypic criterion and one etiologic criterion.

### 2.5 Model training and performance evaluation

Using random sampling techniques, patients were divided into a training set and a validation set at a ratio of 7:3. Four representative supervised machine learning algorithms were employed to construct predictive models: logistic regression (LR), support vector machine (SVM), light gradient boosting machine (LGBM), and extreme gradient boosting (XGBoost). These algorithms were selected for their demonstrated effectiveness in handling structured clinical datasets and their complementary strengths in model performance.

The evaluation of the model’s performance is conducted based on three essential indicators: model discrimination, calibration, and clinical utility. After identifying the optimal model, techniques such as SHAP summary analysis, SHAP dependence analysis, and SHAP force analysis were utilized to conduct a comprehensive examination of the model’s output results from both global and local perspectives.

### 2.6 Statistical analysis

Data analysis was performed via R software, version 4.4.2. Continuous variables are summarized as the means with standard deviations or medians with interquartile ranges, depending on the distribution of the data. Continuous variables were compared via either an independent-sample t test for normally distributed data or a Mann–Whitney U test for nonnormally distributed data. Categorical variables are presented as counts and percentages, with group comparisons made via either a chi-square test or Fisher’s exact test.

### 2.7 Ethical considerations

All procedures involving human participants were conducted according to the ethical standards outlined in the 1964 Helsinki Declaration and its subsequent amendments or equivalent ethical guidelines. Ethical approval for the study, including the acquisition of information from electronic medical records, was granted by the West China Hospital of Stomatology Ethics Committee (Grant Number: WCHSIRB-D-2024-107). Informed consent was obtained from all participants before their inclusion in the study.

## 3 Results

### 3.1 Characteristics of the study sample

A total of 487 oral cancer patients were included in this study, of whom 251 (51.54%) were identified as experiencing postoperative malnutrition, whereas 236 (48.46%) did not exhibit signs of malnutrition. The cohort included 282 males (58.0%) and 205 females (42.0%), with a median age of 61 years. For model development and validation, the study population was divided into a training set of 340 patients and a validation set of 147 patients. The distributions of malnourished and normally nourished patients were comparable between the two sets: 174 (51%) malnourished versus 166 (49%) normally nourished in the training set and 77 (52%) malnourished versus 70 (48%) normally nourished in the validation set. The detailed demographic and clinical characteristics of these subsets are presented in **Table 1**.

**Table 1:** Comparison of the training set and validation set.

Variables	Validation set (n = 147)			Training set (n = 340)			*P Value*
	Normal nutrition (n = 70)	Malnutrition (n = 77)	*P Value*	Normal nutrition (n =166)	Malnutrition (n = 174)	*P Value*	
Age	60.76 ± 13.28	59.87 ± 12.64	0.680	59.5 (50.25, 69)	61 (55, 69.75)	0.065	0.994
BMI	24.2 (22.33, 26.95)	23.4 (21.4, 25.3)	0.102	23.8 (22.22, 25.9)	23.55 (21.9, 25.37)	0.284	0.923
Erythrocyte	4.5 ± 0.48	4.36 ± 0.58	0.108	4.45 ± 0.47	4.4 ± 0.53	0.306	0.981
Leukocyte, Median (Q1,Q3)	6 (5.15, 7.23)	5.22 (4.31, 5.95)	< 0.001	6.01 (5.15, 7.14)	5.71 (4.64, 7.01)	0.065	0.143
Neutrophil, Median (Q1,Q3)	3.4 (2.78, 4.49)	3.13 (2.59, 3.78)	0.135	3.4 (2.73, 4.34)	3.54 (2.73, 4.52)	0.515	0.083
Monocyte, Median (Q1,Q3)	0.46 (0.37, 0.58)	0.43 (0.3, 0.5)	0.081	0.46 (0.35, 0.58)	0.44 (0.34, 0.58)	0.801	0.471
Lymphocyte, Median (Q1,Q3)	1.81 (1.58, 2.27)	1.37 (1.14, 1.7)	< 0.001	1.81 (1.55, 2.15)	1.38 (1.16, 1.78)	< 0.001	0.641
HB, Mean ± SD	135.09 ± 15.34	132.06 ± 16.27	0.249	135.31 ± 14.14	133.95 ± 15.34	0.396	0.468
PLT, Median (Q1,Q3)	202.5 (166, 249.75)	182 (130, 227)	0.038	199.5 (169, 243)	190.5 (151, 231.75)	0.057	0.322
ALB, Median (Q1,Q3)	42.95 (41.52, 44.18)	42.5 (40.4, 44.3)	0.252	42.87 ± 2.6	42.32 ± 3.3	0.030	0.958
GLB, Median (Q1,Q3)	26.88 (24.18, 30.15)	25.64 (23.02, 28.4)	0.045	26.23 (24.1, 28.88)	26.54 (23.64, 29)	0.767	0.844
TP, Median (Q1,Q3)	70.32 (67.23, 73.4)	68.06 (64.42, 71.02)	0.019	69.38 ± 4.61	68.53 ± 5.39	0.162	0.746
CRP, Median (Q1,Q3)	1.5 (0.8, 2.5)	1.1 (0.7, 3.1)	0.155	1.2 (0.8, 3.45)	1.4 (0.8, 5.47)	0.092	0.351
TC, Mean ± SD	4.75 ± 0.98	4.54 ± 0.94	0.179	4.82 ± 0.92	4.49 ± 0.97	0.002	0.832
Gender, n (%)			0.068			0.001	0.940
Female	35 (50)	26 (34)		86 (52)	58 (33)		
Male	35 (50)	51 (66)		80 (48)	116 (67)		
T stage, n (%)			0.211			< 0.001	0.173
T_1-2_	40 (57)	35 (45)		91 (55)	58 (33)		
T_3-4_	30 (43)	42 (55)		75 (45)	116 (67)		
Tumor factors Site, n (%)			0.038			0.051	0.133
Tongue	31 (44)	28 (36)		87 (52)	63 (36)		
Gingiva	8 (11)	13 (17)		28 (17)	41 (24)		
Cheek	20 (29)	22 (29)		36 (22)	51 (29)		
Hard palate	8 (11)	2 (3)		6 (4)	6 (3)		
Mouth floor	3 (4)	12 (16)		9 (5)	13 (7)		
Cervical lymphatic dissection, n (%)			0.012			0.029	0.412
No	31 (44)	18 (23)		58 (35)	41 (24)		
Yes	39 (56)	59 (77)		108 (65)	133 (76)		
Repair and reconstruction, n (%)			0.020			0.001	0.631
No	32 (46)	20 (26)		69 (42)	42 (24)		
Yes	38 (54)	57 (74)		97 (58)	132 (76)		
Smoking, n (%)			0.122			0.12	0.769
No	47 (67)	41 (53)		110 (66)	100 (57)		
Yes	23 (33)	36 (47)		56 (34)	74 (43)		
Alcohol, n (%)			0.435			0.217	0.754
No	48 (69)	47 (61)		110 (66)	103 (59)		
Yes	22 (31)	30 (39)		56 (34)	71 (41)		
Diabetes, n (%)			0.688			0.004	0.089
No	65 (93)	69 (90)		151 (91)	138 (79)		
Yes	5 (7)	8 (10)		15 (9)	36 (21)		
Hypertension, n (%)			0.556			1	0.508
No	44 (63)	53 (69)		104 (63)	108 (62)		
Yes	26 (37)	24 (31)		62 (37)	66 (38)		
Recurrence, n (%)			1			0.744	1
No	62 (89)	69 (90)		146 (88)	156 (90)		
Yes	8 (11)	8 (10)		20 (12)	18 (10)		
Tracheotomy, n (%)			1			0.202	0.380
No	69 (99)	75 (97)		162 (98)	164 (94)		
Yes	1 (1)	2 (3)		4 (2)	10 (6)		

BMI, body mass index; HB, hemoglobin; PLT, platelets; ALB, albumin; GLB, globulin; TP, total protein; CRP, C-reactive protein; TC, total cholesterol.

### 3.2 Univariate and multivariate regression analyses

In the training set, predictors were initially analyzed via univariate logistic regression. Variables with statistical significance (*p* < 0.1) were subsequently incorporated into a stepwise multivariate regression model. Based on the Akaike Information Criterion (AIC), the predictive factors to be included in the model were selected. This criterion aims to minimize the AIC, allowing variables with *P* > 0.05 to be included, thereby ensuring the best balance between model complexity and goodness of fit. Ultimately, seven characteristic variables were identified as the key predictive factors for constructing the machine learning model. These variables included sex, T stage, repair and reconstruction, diabetes status, age, lymphocyte count, and total cholesterol (TC) level, as presented in **Table 2**.

**Table 2:** Univariate and multivariate logistic regression analyses.

Variables	Univariate logistic		Multivariate logistic	
	OR (95%CI)	*P*	OR (95%CI)	*P*
Age	1.017(0.999-1.035)	0.063	1.017 (0.996-1.038)	0.101
Lymphocyte	0.232(0.141-0.368)	< 0.001	0.226 (0.133-0.370)	< 0.001
ALB	0.939(0.871-1.009)	0.094		
CRP	1.033(1.009-1.063)	0.014		
TC	0.692(0.545-0.871)	0.002	0.761 (0.585-0.986)	0.04
Gender				
Female	Ref.	_	Ref.	_
Male	2.15(1.391-3.344)	0.001	2.025 (1.229-3.360)	0.006
T stage				
T_1-2_	Ref.	_	Ref.	_
T_3-4_	2.427(1.569-3.78)	< 0.001	1.781 (1.039-3.065)	0.036
Tumor factors Site				
Tongue	Ref.	_		
Gingiva	2.022(1.138-3.637)	0.017		
Cheek	1.956(1.149-3.361)	0.014		
Hard palate	1.381(0.414-4.606)	0.591		
Mouth floor	1.995(0.811-5.108)	0.137		
Cervical lymphatic dissection				
No	Ref.	_		
Yes	1.742(1.088-2.81)	0.022		
Repair and reconstruction				
No	Ref.	_	Ref.	_
Yes	2.236(1.41-3.577)	0.001	1.881 (1.057-3.375)	0.032
Smoking				
No	Ref.	_		
Yes	1.454(0.937-2.263)	0.096		
Diabetes				
No	Ref.	_	Ref.	_
Yes	2.626(1.403-5.138)	0.003	2.106 (1.036-4.451)	0.044

ALB, Albumin; CRP, C-reactive protein; TC, Total cholesterol; OR, Odds ratio; CI, Confidence interval.

### 3.3 Comparative analysis of multiple models and validation of the optimal model

After the required feature variables for modeling were selected, four supervised machine learning algorithms, namely, LR, SVM, LGBM, and XGBoost, were employed to analyze the training set data. The predictive performance of the four models was thoroughly assessed from three perspectives: discrimination, calibration, and clinical utility.

**Figure 2:**
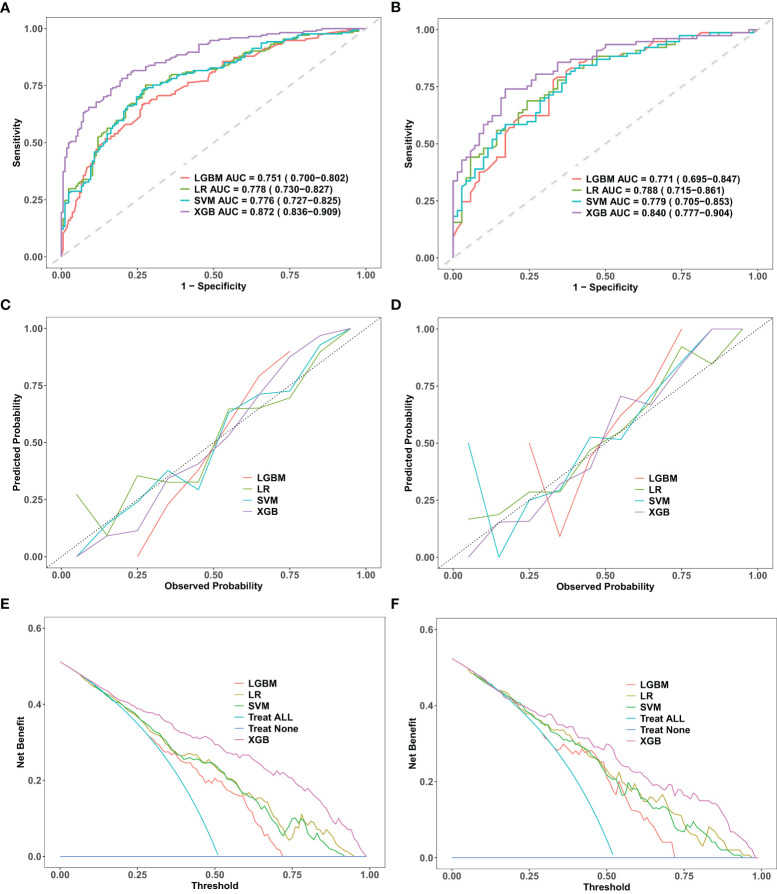
Comparative performance evaluation of machine learning models. Performance evaluation of four machine learning models—logistic regression (LR), support vector machine (SVM), light gradient boosting machine (LGBM), and extreme gradient boosting (XGBoost)—in the training and validation datasets. **(A, B)**: ROC curves with AUC values, showing XGBoost’s superior discriminative performance. **(C, D)**: Calibration curves indicating a strong fit and robust generalization for XGBoost. **(E, F)**: DCA curves demonstrating XGBoost’s net clinical benefit. ROC, receiver operating characteristic; DCA, decision curve analysis; AUC, area under the curve; LR, logistic regression; SVM, support vector machine; LGBM, light gradient boosting machine; XGBoost, extreme gradient boosting.

Discrimination refers to the model’s capacity to accurately classify patients into low-risk and high-risk categories. In this study, a variety of indicators were employed to assess the classification performance of the model, including the receiver operating characteristic (ROC) curve, area under the curve (AUC), accuracy, specificity, precision, sensitivity, and F1 score. Based on the ROC curves of four different machine learning prediction models, the XGBoost model achieves the highest performance, with an AUC of 0.872 [95% confidence interval (CI): 0.836−0.909] on the training set and 0.840 (95% CI: 0.777–0.904) on the validation set (**Figures 2A, B**). **Table 3** provides a comprehensive analysis of the discrimination evaluation metrics for four machine learning models, evaluated on both the training set and the validation set.

**Table 3:** Model discrimination assessment.

	Model	AUC	Accuracy	Specificity	Precision	Sensitivity	F1-score
Training	LR	0.778	0.738	0.723	0.740	0.753	0.746
	XGB	0.872	0.791	0.801	0.805	0.782	0.793
	SVM	0.776	0.732	0.735	0.743	0.730	0.736
	LGBM	0.751	0.701	0.735	0.725	0.667	0.695
Validation	LR	0.788	0.705	0.657	0.707	0.753	0.730
	XGB	0.840	0.756	0.771	0.781	0.740	0.760
	SVM	0.779	0.692	0.657	0.700	0.727	0.713
	LGBM	0.771	0.668	0.686	0.694	0.649	0.671

AUC, area under the curve; LR, logistic regression; SVM, support vector machine; LGBM, light gradient boosting machine; XGBoost, extreme gradient boosting.

The calibration curve is employed to evaluate the consistency between the actual occurrence probabilities of postoperative malnutrition outcomes in oral cancer patients, as observed in both the training and validation sets, and the predicted probabilities generated by various models. Among these models, the calibration curve for the XGBoost model closely aligns with the ideal line, indicating that this model demonstrates the highest degree of correspondence between its predicted probabilities and actual outcomes (**Figures 2C, D**).

Under the condition of equivalent diagnostic efficacy, decision curve analysis (DCA) conducted on the training and validation sets of XGBoost provided a substantial net benefit for clinical decision-making across a wide range of threshold probabilities (**Figures 2E, F**). In the validation set, when the prediction threshold was established within the range of 0.2 to 0.98, the DCA results for the XGBoost model did not intersect with either of the two extreme curves. This finding suggests that, in comparison to strategies involving complete intervention or total non-intervention, the XGBoost model is capable of delivering greater net benefits across a wider spectrum of thresholds. Consequently, this further underscores its potential advantages in clinical practice. Therefore, we conclude that the XGBoost algorithm is the optimal model for this dataset, offering strong predictive accuracy and clinical applicability.

### 3.4 Model explanation and clinical significance analysis

To clarify the clinical significance of the XGBoost model, we used the SHAP method to elucidate its prediction process and results. Identify and elucidate the significance of predictive features through SHAP to improve its applicability in clinical decision-making. Two levels of explanations were provided: global explanations, which describe the overall functionality of the model, and local explanations, which interpret individual predictions.

**Figure 3:**
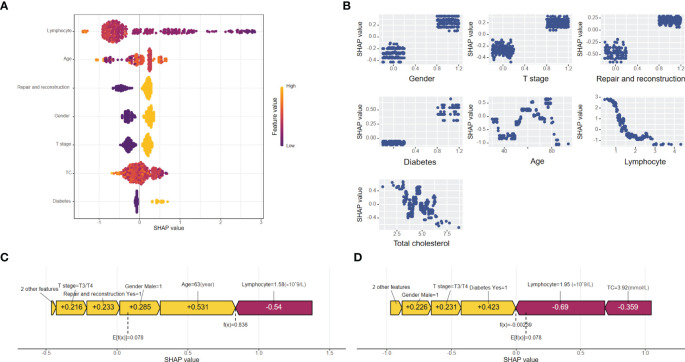
**(A)** Importance chart of the SHAP variables. The SHAP beeswarm plot ranks feature importance in the XGBoost model on the basis of average SHAP values. Lymphocyte count, age, and repair and reconstruction are the top predictors of postoperative malnutrition, with feature values indicated by color gradients (yellow for high, purple for low). **(B)** Dependence plot of the SHAP variables. The SHAP dependence plot delineates the marginal effects of seven features on the predictive outcomes of the machine learning model, emphasizing the relationship between malnutrition and its predictors. **(C)** SHAP force plot for a "true positive" patient. SHAP force plot for a "true positive" patient with a SHAP value of 0.836, above the baseline (E[f(x)] = 0.078). Positive contributions (yellow) from age, sex, and repair and reconstruction drive the prediction of malnutrition, whereas negative contributions (red) from lymphocyte levels reduce it. **(D)** SHAP force plot for a "true negative" patient. SHAP force plot for a "true negative" patient with a SHAP value of 0.00239, below the baseline (E[f(x)] = 0.078). Negative contributions (red) from lymphocyte levels and total cholesterol drive the prediction of no malnutrition, with minor positive contributions (yellow) from diabetes and sex.

The global explanation, illustrated by the SHAP summary in **Figure 3A**. The Bee Warm Map, provided by SHAP, is a visualization tool that illustrates the significance and influence of variables in a dataset. Each point on the map represents the SHAP value for a specific sample, allowing analysis of variable importance, effect directionality, and predictive accuracy. The Y-axis shows the average impact of these variables on model predictions, with rows organized for better visibility. The most critical variables are at the top. For the XGBoost postoperative malnutrition risk prediction model for oral cancer, feature importance ranks as follows: lymphocytes, age, free flap transplantation repair surgery, gender, tumor T stage, total cholesterol, and history of diabetes. The X-axis displays SHAP values quantifying each variable’s contribution to model predictions.

**Figure 3B** shows a SHAP dependence plot that visualizes the impact of seven predictors on the XGBoost model’s predictions. The X-axis represents feature values, while the Y-axis displays SHAP values, which quantify each feature’s significance in relation to the prediction outcome. This plot clearly indicates whether a predictor has a positive or negative effect on predictions. A positive SHAP value suggests an increased likelihood of postoperative malnutrition, whereas a negative value indicates a decreased probability.

Local explanations were visualized via SHAP force plots to analyze individual patient predictions. In the XGBoost model, E[f(x)] represents the average predicted value and serves as the SHAP reference. Higher SHAP values indicate a greater likelihood of postoperative malnutrition. The color bar in the force plots reflects the feature contribution intensity: red (left arrow) signifies a negative impact (reduced SHAP value), whereas yellow (right arrow) denotes a positive impact (increased SHAP value). **Figure 3C** shows a “true positive” patient with a SHAP value of 0.836, above baseline, indicating malnutrition. **Figure 3D** depicts a “true negative” patient with a SHAP value of 0.00239, below baseline, indicating no malnutrition.

### 3.5 Implementation of the web calculator

**Figure 4:**
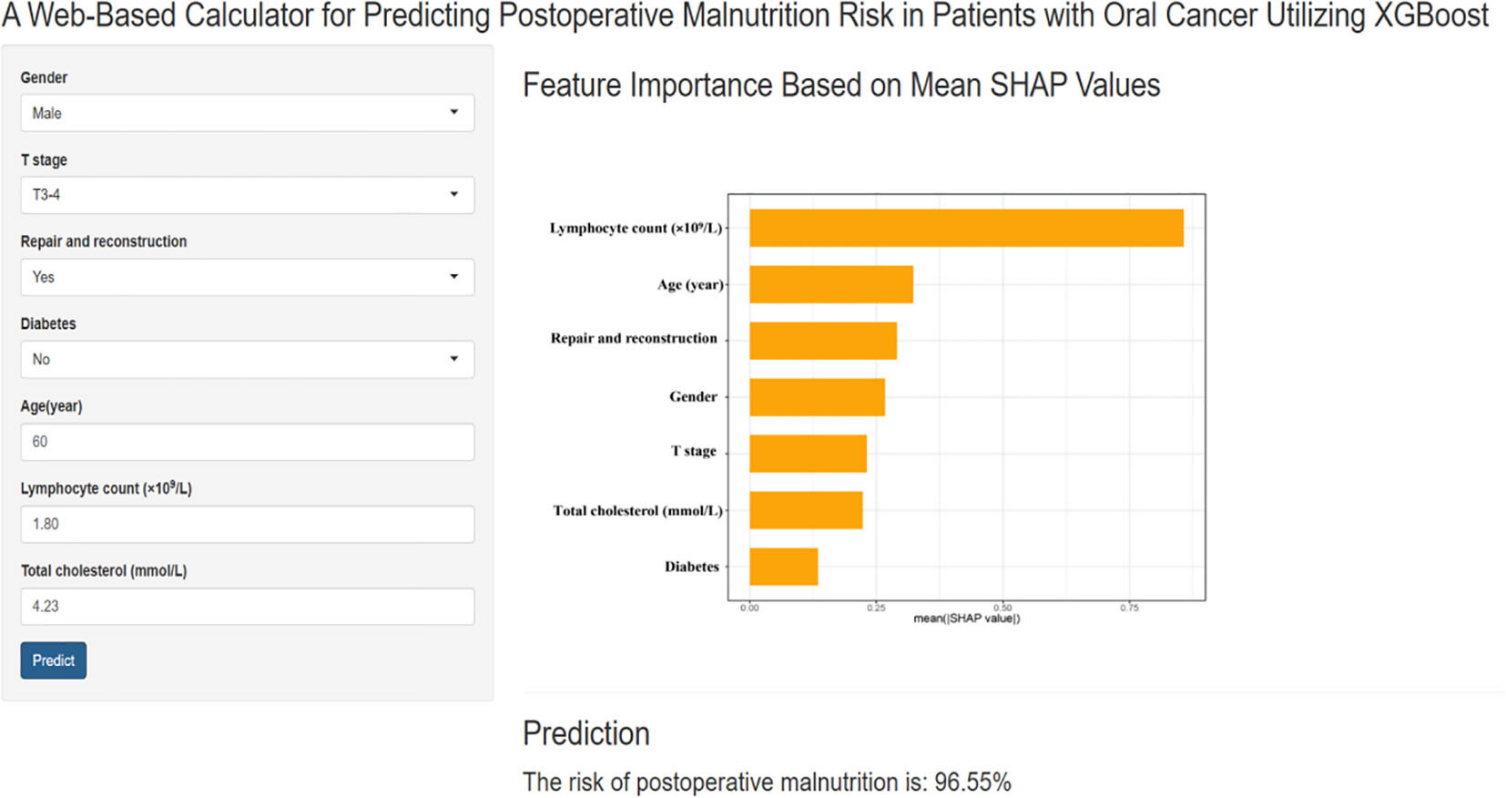
A website-based tool for predicting postoperative malnutrition risk in oral cancer. Web-based tool for predicting postoperative malnutrition risk in oral cancer patients. The application, developed via the final prediction model, allows users to input the seven required features and automatically predicts the risk of malnutrition. The feature importance is displayed on the basis of the mean SHAP values, and the prediction result is shown with a calculated malnutrition risk of 96.95%.

As shown in **Figure 4**, the final prediction model was deployed as a web-based application to facilitate its use in clinical settings. By entering the actual values of the 7 features required for the model, the application automatically predicts the risk of postoperative malnutrition in individual patients with oral cancer. The web application is accessible online at the following link: https://pred-mod.shinyapps.io/XGBoost/.

## 4 Discussion

Malnutrition significantly impacts both survival rates and quality of life in patients with oral cancer, serving as a major contributor to postoperative complications and mortality (19, 20). Previous studies have reported that the incidence of postoperative malnutrition in patients with oral cancer ranges from 37.3% to 60.68% (21, 22), findings that closely align with the results of this study. These data suggest that individuals with oral cancer are particularly vulnerable to malnutrition and face an elevated risk of developing nutritional deficiencies following surgical intervention. Therefore, clinical practitioners need to strengthen dynamic screening and assessment of nutritional risk in postoperative patients, ensuring timely identification of at-risk individuals and providing adequate nutritional support (23).

The traditional methods for nutritional screening are based on complex scales. While these approaches are beneficial, they contribute to an increased clinical workload and can only evaluate a patient’s current nutritional status without forecasting the risk of postoperative malnutrition (22, 24). This study utilizes the GLIM diagnostic criteria as a tool to assess postoperative malnutrition outcomes in patients with oral cancer, and it develops a risk prediction model employing machine learning algorithms. The findings indicate that the XGBoost model exhibits optimal performance, demonstrating high clinical utility by effectively predicting the likelihood of postoperative malnutrition in oral cancer patients. This provides a crucial foundation for the early identification of high-risk individuals and the implementation of targeted nutritional interventions. Therefore, developing and integrating predictive tools into clinical practice holds significant potential for enhancing patient outcomes and reducing the burden of malnutrition in this high-risk population.

In this study, we developed and evaluated four ML models for predicting postoperative malnutrition in patients with oral cancer. The models were assessed on the basis of their discrimination ability, accuracy, and clinical applicability. Ultimately, we successfully developed and validated a robust ML-based predictive model for postoperative malnutrition. This model utilized only preoperative and intraoperative data, which are readily accessible, allowing for the early identification of patients at risk of surgical malnutrition. We employed the SHAP method to generate feature density scatter plots and importance rankings on the basis of SHAP values to increase the interpretability and usability of the model in clinical settings. These visualizations clarify the contributions of individual features to the risk of malnutrition. SHAP force plots further demonstrated the predictive results for individual patients in the training set, making the model more transparent and clinically relevant. Additionally, we developed a user-friendly website-based application to support the practical implementation of this tool, enabling clinicians to assess the risk of postoperative malnutrition and guide interventions efficiently.

The XGBoost model developed in this study incorporated seven key predictive variables: lymphocyte count, age, repair and reconstruction status, sex, T stage, total cholesterol, and diabetes status. Among these factors, the lymphocyte count has emerged as a crucial indicator of nutritional status and immune function and serves as a prognostic factor in head and neck cancer patients because of its role in antitumor and immune responses (25, 26). Aging further exacerbates malnutrition risk by impairing physiological and immune functions, making elderly oral cancer patients particularly vulnerable to postoperative malnutrition (27), a finding that is consistent with prior research (21, 28). Additionally, surgical trauma from tumor resection and subsequent repair significantly compromise eating function, prolong recovery, and reduce oropharyngeal efficiency, contributing to the risk of malnutrition (29, 30). Our findings highlight the need for targeted nutritional support for high-risk patients undergoing surgical treatment (23, 31). Furthermore, sex differences play a role in malnutrition risk, with male patients showing greater susceptibility than females do, which is consistent with previous research (32–34). The T stage of a tumor reflects its development, with advanced stages associated with larger tumors, more extensive surgeries, and an increased risk of malnutrition. Thus, the T stage independently affects both the postoperative prognosis and nutritional status of oral cancer patients (28, 35).

Additionally, our study revealed a significant association between low preoperative total cholesterol levels and postoperative malnutrition. This may be due to cholesterol’s role in cell membrane structure, signal transduction, nutrient absorption, glucose metabolism, and stress response (36, 37). Previous studies indicate that cholesterol metabolism affects dietary intake (38); individuals with higher preoperative total cholesterol have a lower risk of postoperative malnutrition (39). Additionally, the preoperative total cholesterol level is associated with the prognosis of oral cancer patients (40), highlighting its potential as an indicator for assessing postoperative nutritional status and predicting clinical outcomes. Diabetes is another critical predictor, as patients with diabetes are more likely to experience stress-related metabolic disruptions following surgery, further increasing their risk of malnutrition (41). These findings highlight the critical need to integrate multiple clinical variables, including preexisting comorbidities, into predictive models to facilitate the early identification of patients at risk of malnutrition (42).

This study conducted a comprehensive literature review to summarize the key influencing factors associated with postoperative malnutrition in patients with oral cancer, as identified in prior research. Given the challenges related to identifying predictive factors, cost-effectiveness, clinical applicability, and operability, we ultimately selected and included 23 potential variables for further analysis. Based on these variables, a survey instrument was developed. Following established guidelines for developing machine learning prediction models (43) and employing appropriate statistical methods, we refined our selection to seven clinically accessible and objectively reliable variables as predictive factors. This refinement facilitated the construction of four distinct machine learning prediction models. Among these models, the XGBoost model exhibited superior performance by effectively predicting the risk of postoperative malnutrition in patients with oral cancer. The model demonstrated excellent and stable predictive capabilities across both training and testing datasets, indicating its robust generalization ability. Utilizing the XGBoost model, we created a web-based calculator designed to predict the risk of postoperative malnutrition in patients with oral cancer. This tool not only aids in early identification of high-risk individuals but also enables precise nutritional interventions and effective clinical risk management strategies.

This study has several limitations. First, the XGBoost model was trained and validated on data from a single center, which may raise concerns regarding its generalizability to other centers or regions. In particular, regional and ethnic variations in nutritional status, dietary habits, healthcare access, and other socio-economic factors can significantly influence the accuracy and applicability of the model. However, the model incorporates objective variables and indicators, enhancing its reliability and potential for broader applicability. Second, the study focused primarily on predicting short-term malnutrition risk in oral cancer patients following surgery, without addressing long-term changes in nutritional status postintervention. To address these limitations, future research should focus on external validation across diverse geographic and ethnic populations to assess the model’s robustness and adaptability. Additionally, adopting a longitudinal design will allow for the monitoring of nutritional status over time post-surgery, enabling a more comprehensive understanding of malnutrition risk. This will help refine the model’s performance and enhance its clinical applicability, providing a strong foundation for future implementation in clinical environments.

## 5 Conclusions

We developed the XGBoost machine learning model to predict the risk of malnutrition in patients with oral cancer following surgery. To enhance interpretability, we employed the SHAP method and created a web interface for ease of use by clinical staff. By leveraging this model to assess malnutrition risk during the early postoperative phase and implementing targeted nutritional strategies, we aim to enhance the postoperative nutritional status of patients diagnosed with oral cancer and to facilitate their recovery.

## Data Availability

The datasets presented in this study can be found in online repositories. The names of the repository/repositories and accession number(s) can be found in the article/supplementary material.
